# 

**Published:** 1861-04

**Authors:** 


New Series, Vol. 4. No. 4. Whole Series, Vol. 18, No. 4.

### THE CHICAGO
### MEDICAL JOURNAL.

DANIEL BRAINARD, M. D.,

J. ADAMS ALLEN, M. D.,

Editors and Proprietors.

APRIL, 1861.

CHICAGO:

WM. PIGOTT, BOOK AND JOB PRINTER, 130 CLARK STREET.

1861.

